# Nano- and Microstructured Systems for Controlled Release of Agricultural Inputs: Innovations for Efficiency and Sustainability

**DOI:** 10.1021/acs.jafc.4c12980

**Published:** 2025-04-24

**Authors:** Aline Rombega Tito Rosa, Renato Farias do Valle Jr., Marcos Vinicius da Silva, Hugo Felix Perini, Carlo José Freire Oliveira, Rodrigo César Rosa, Antonio Carlos Shimano, Anielle Christine Almeida Silva, Luís Carlos de Morais

**Affiliations:** † Network of Translational Nanobioplatforms, Federal University of Triângulo Mineiro Mineira (UFTM), Vigário Carlos Street, 100, CEP, Abadia, 38025-350 Uberaba, Minas Gerais, Brazil; ‡ Geoprocessing Laboratory, Federal Institute of Triângulo Mineiro (IFTM), João Batista Ribeiro Street, 4000 - Distrito Industrial II, 38064-790 Uberaba,Minas Gerais, Brazil; § Physics Institute, Federal University of Alagoas, Lourival Melo Mota Avenue, s/n, CEP, Tabuleiro do Martins, 57072-900 Maceió, Alagoas, Brazil

**Keywords:** agricultural systems, nanotechnology, biodegradable polymers, soil remediation

## Abstract

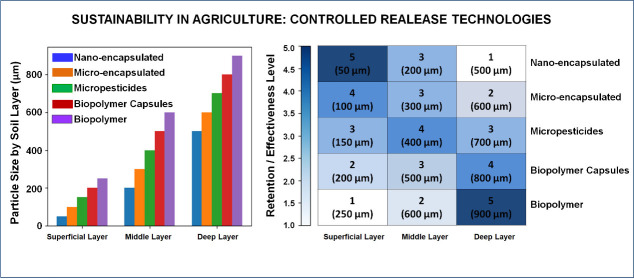

Nano and microstructured systems for the controlled release of agricultural inputs represent a significant advancement in sustainable agriculture. These technologies enable the encapsulation of nutrients and pesticides, ensuring gradual and targeted delivery while reducing waste and enhancing plant absorption. Biodegradable materials, such as chitosan and alginate, offer eco-friendly solutions that improve efficiency under challenging conditions, including salinity and drought. Recent innovations have led to increased crop productivity, reduced pesticide application, and improved soil remediation. For example, nanoparticles can adsorb heavy metals like cadmium and lead, facilitating the restoration of contaminated soils. Despite these benefits, challenges remain, including the need for clear regulatory frameworks and further research on the long-term ecological impacts of nanomaterials. This review highlights the critical role of nano and microstructured systems in advancing agricultural sustainability. By bridging technological innovation with practical applications, these systems have the potential to transform global farming, making it more efficient, resilient, and environmentally sustainable.

## Introduction

Controlled release systems for nutrients and pesticides based on nano and microstructures represent a significant advancement for sustainable agribusiness, enhancing input efficiency while reducing environmental impacts. The use of nanotechnologies and microstructures enables the encapsulation of active substances, such as essential nutrients and agricultural pesticides, facilitating a gradual and targeted release. This controlled release prevents the leaching of compounds, reduces the frequency of applications, and promotes more effective plant absorption, minimizing waste and improving agricultural productivity.^1−3^

Recent studies highlight the use of biodegradable materials, such as chitosan and alginate, in nutrient encapsulation, providing environmentally safe solutions while increasing the durability of active compounds in the soil.^4,5^ Chitosan, for example, is widely used for its biocompatible and antimicrobial properties, as well as its ability to protect nutrients from rapid degradation, creating a more stable environment for root absorption.^6^ These biopolymers enable the efficient encapsulation of both nutrients and pesticides, improving plants’ ability to withstand adverse conditions such as low water availability or high soil salinity.^7^

The innovation brought by nanofertilizers and encapsulated pesticides is also reflected in bioremediation, an approach that uses nanoparticles to remove contaminants, such as heavy metals, from agricultural soils, recovering degraded areas and increasing their viability for planting. Research demonstrates that these particles can adsorb toxic elements, such as cadmium and lead, rehabilitating contaminated soils for agricultural use, representing a positive impact on environmental conservation and food security.^8,9^

The introduction of nano and microstructured encapsulation systems for the controlled release of pesticides and nutrients not only enhances the precision and efficiency of input use but also promotes more sustainable practices. This is particularly relevant in the current agricultural scenario, where the demand for food production continues to grow, while alternatives are sought to mitigate the environmental impacts of conventional practices.^10,11^ The combination of these technologies enhances crop productivity and quality while significantly reducing environmental impacts. By minimizing pesticide use, these systems directly contribute to lower contamination of water and soil resources. Additionally, the controlled release of inputs reduces leaching and runoff, further mitigating pollution risks.^12,13^

However, challenges remain, particularly concerning the safety and regulation of these new technologies. The long-term toxicity of nanomaterials and the need for in-depth studies to understand their interactions with the environment and nontarget organisms are pressing issues. Researchers have emphasized the importance of adequate regulations to ensure that the benefits of these technological advancements are fully realized without compromising environmental safety and human health.^14,15^

Thus, adopting nano and microstructured systems for controlled release in agribusiness represents an innovation with the potential to transform agriculture, enabling more efficient resource use and more sustainable production. This study aims to identify and synthesize the main advancements and challenges of encapsulation technologies for the controlled release of nutrients and pesticides. The focus is on evaluating these studies’ contributions to agribusiness sustainability and identifying emerging trends and research gaps.

## Materials and Methods

### Protocol and Registration

This systematic review was conducted following the Preferred Reporting Items for Systematic Reviews and Meta-Analysis Protocol guidelines (PRISMA-P).^16^ The protocol was based on methodologies used in previous risk assessment studies, adapted to the investigation of nano and microstructured systems for controlled release of nutrients and pesticides in agriculture.^17,18^ The methodology was detailed to allow replication, including all steps adopted in the planning, execution, and data analysis processes.

### Eligibility Criteria

The eligibility criteria for the studies were defined using the PECOS framework, ensuring a rigorous selection of articles relevant to nano and microstructured systems for controlled release of nutrients and pesticides in agribusiness. The criteria are detailed as follows:

- Population (P): Studies investigating the application of nano and microstructured systems in agricultural environments, focusing on controlled release of nutrients and pesticides.
- Exposure (E): Research related to the development and practical use of controlled release systems, from encapsulation to implementation in crops.
- Comparison (C): Comparison between conventional systems for releasing agricultural inputs and nano and microstructured technologies.
- Outcomes (O): Studies evaluating measurable impacts, such as increased input efficiency, reduced pesticide use, mitigated environmental impacts, and improved agricultural sustainability.
- Study Type (S): Original articles and reviews, including experimental studies, case studies, and systematic reviews providing empirical data or robust analyses.

### Inclusion Criteria

1. Development and Practical Application: Studies presenting the formulation, development, or practical application of nano and microstructured systems for the controlled release of agricultural inputs.
2. Experimental Data: Articles with field or laboratory experimental data measuring system efficiency, such as nutrient or pesticide release rates, agricultural productivity impact, or reduction of environmental contaminants.
3. Language and Time Frame: Publications in English or Portuguese published since 2010.

### Exclusion Criteria

To ensure the inclusion of relevant and robust studies, the following exclusion criteria were detailed:

1. Theoretical Studies Without Practical Application: Studies lacking experimental validation or practical analysis of described systems.
2. Absence of Quantitative Analysis: Studies without detailed and measurable quantitative analyses related to the efficiency or environmental impact of nano and microstructured systems.

- Accepted Metrics: Nutrient or pesticide release rates over time; Half-life of inputs in soil or plants; Quantity of input used per hectare compared to conventional methods; Reduction in environmental contaminants, such as chemical residues in surface or groundwater (mg/L) and soil residues (mg/kg); Impact on greenhouse gas emissions (e.g., CO_2_ equivalent).
- Sustainability Indicators: Productivity increases per hectare; Operational cost reductions; Soil quality improvements, measured through indicators such as increased organic matter or nutrient retention capacity.

3. Lack of Experimental Validation: Studies lacking results from controlled or field experiments supported by replicates and the presentation of confidence intervals or standard errors.
4. Lack of Statistical Analysis: Comparative studies not presenting robust statistical tests, such as ANOVA, *t* tests, or predictive multivariate models.
5. Incomplete Texts: Reviews, conference abstracts, opinions, and case reports without full-text availability or reproducible data.

### Information Sources and Search Strategy

The search was conducted in academic databases, including Scopus, Web of Science, and ScienceDirect, using a search strategy that combined descriptors and Boolean operators in English, such as ″nanostructured″ AND ″controlled release″ AND ″agriculture″ and ″microstructures″ AND ″nutrients″ AND ″pesticides″ AND ″efficiency.″ To enhance coverage, open repositories like Google Scholar were consulted for gray literature (e.g., dissertations and technical reports).

Manual searches and the exploration of references from included articles were performed to ensure the inclusion of relevant studies. Experts in the field were also consulted to identify additional articles and validate the review’s comprehensiveness.

### Study Selection

Two independent reviewers initially evaluated titles and abstracts to verify eligibility criteria. After this initial screening, full texts were analyzed and thoroughly assessed for inclusion criteria. In case of disagreements, a third reviewer was consulted for resolution.

### Data Collection

Extracted data included: general study characteristics (authors, year, location), type of nano or microstructured system used, experimental methodology, main results regarding efficiency and sustainability, and environmental impact. Data extraction was conducted independently by two reviewers, and any discrepancies were resolved by a third evaluator.

### Risk of Bias Assessment

The risk of bias in the studies was assessed using the Joanna Briggs Institute (JBI) Critical Appraisal Tool, which provides a framework for evaluating the methodological quality of case-control, cohort, and cross-sectional studies. Each study was classified as having “high,” “moderate,” or “low” risk of bias, depending on the score obtained based on established criteria.^19^

## Results

### Study Selection Flowchart (PRISMA)

**Figure 1:**
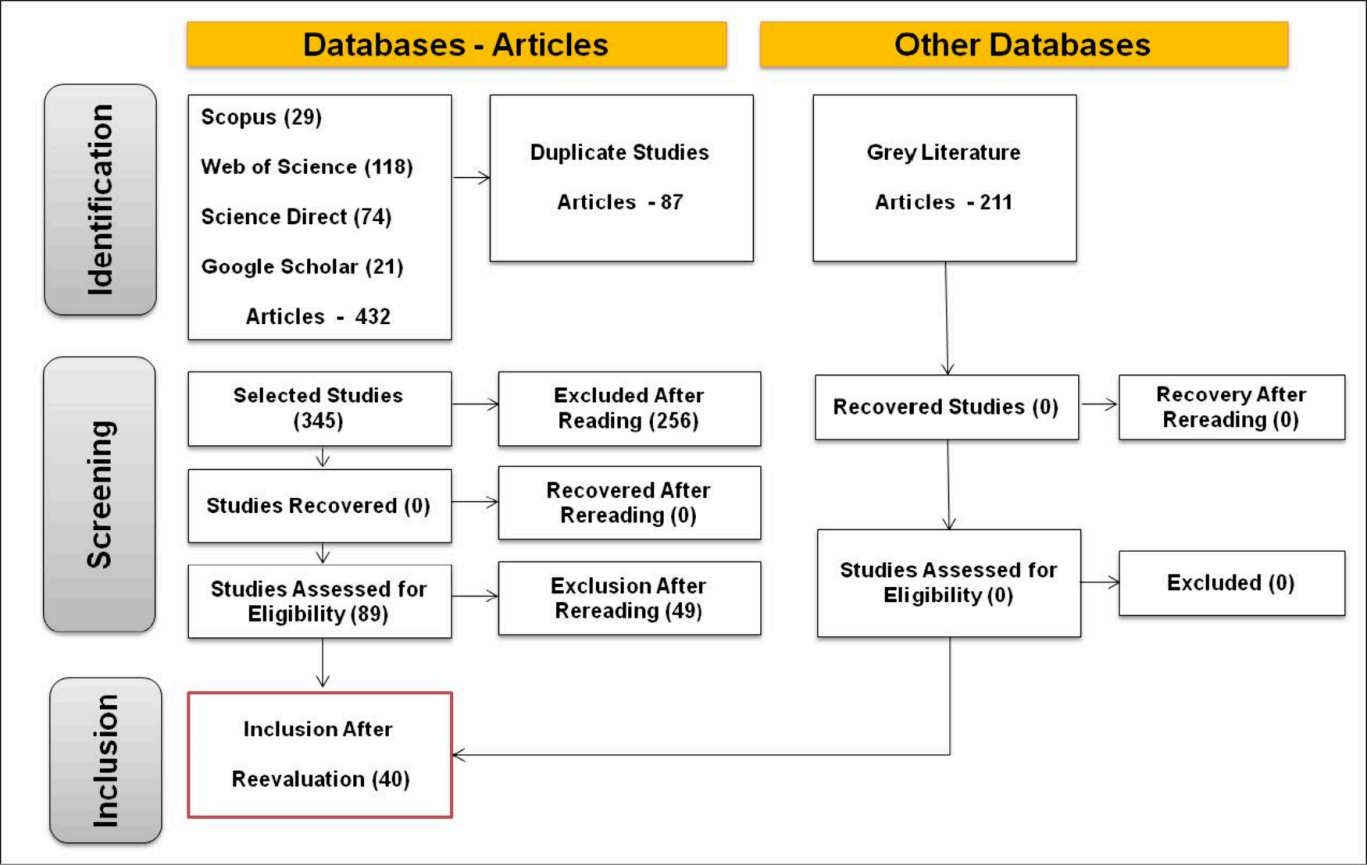
PRISMA flowchart detailing the article selection process.

The database search across Scopus, Web of Science, ScienceDirect, and Google Scholar initially identified 432 records. After removing 87 duplicates, 345 articles were screened based on titles and abstracts. From this screening, 256 studies were excluded, with 172 removed due to a lack of direct relevance to the topic and 84 due to the absence of pertinent experimental data. Subsequently, 89 articles underwent full-text evaluation, of which 40 met all eligibility criteria and were included in the final analysis. The complete selection process is detailed in the flowchart presented in Figure 1.

### Description of Studies

The systematic search resulted in an initial 432 records. After duplicate removal and applying eligibility criteria, 40 studies were included in the final analysis. These articles were grouped into four main categories based on the methodologies employed and objectives of the studies: controlled nutrient release, pesticide encapsulation, bioremediation of contaminated soils, and environmental impacts/sustainability. Tables 1, 2, 3, and 4 present the details of the articles reviewed in each group.

The analysis of the studies revealed that most publications occurred between 2015 and 2023, with a geographic concentration of studies conducted in Asia (40%), Europe (30%), and North America (20%). These findings highlight the leadership of these regions in the development and application of encapsulation and controlled release technologies in agribusiness.

The analyzed articles were organized into four main categories according to their objectives and central themes. Most studies (50%) addressed controlled nutrient release, demonstrating a predominant interest in improving the efficiency of agricultural inputs. Following this, 30% of the studies investigated pesticide encapsulation, focusing on reducing negative impacts and increasing precision in pest control. Other prominent areas included the bioremediation of contaminated soils, accounting for 15% of publications, and environmental impacts and sustainability, which, although less frequent, represented 5% of the analyzed articles.

### Controlled Nutrient Release

Studies on controlled nutrient release represented the largest number of publications (40% of the total). This line of research focuses on the application of nano and microstructured systems to increase fertilizer efficiency and minimize losses. Mehra et al. (2021) reported a reduction in the need for fertilizer reapplication in field trials,^1^ while Qadir et al. (2018) highlighted the use of biodegradable polymers, such as chitosan, for gradual release, resulting in lower environmental impact.^4^ Furthermore, Malik et al. (2019) demonstrated a significant reduction in leaching with nanostructured systems.^12^

These findings are particularly relevant for poor soils or those subject to environmental stresses, such as high salinity or low fertility.^14,20^ Detailed data for these studies are available in Table 1.

**Table 1:** Key Studies on Controlled Nutrient Release

author (year)	focus of the study	methodology	key findings
Mehra et al. (2021)	nutrient release	field trials	30% reduction in fertilizer reapplication
. (2018)	use of biodegradable polymers	laboratory tests	controlled release and reduced environmental impact
Malik et alQadir et al. (2019)	controlled nutrient release	tests with nanostructured polymers	significant reduction in leaching losses
Ahmed et al. (2020)	nanofertilizers for poor soils	laboratory trials	improved nutrient bioavailability in deficient soils
Wang et al. (2020)	nanofertilizers in saline soils	greenhouse trials	40% increase in resistance to water stress
Rahman et al. (2020)	nanofertilizers in arid environments	field trials	35% increase in agricultural productivity under low fertility conditions
Singh et al. (2020)	nanotechnology in agribusiness	review of practical applications	increased productivity with reduced environmental impacts
Saifullah et al. (2019)	sustainability in agriculture	review of encapsulation methods	highlighted the use of biodegradable polymers such as chitosan
Li et al. (2021)	encapsulated nutrients	controlled release simulations	gradual control and 25% increase in nutrient absorption by plants

**Figure 2:**
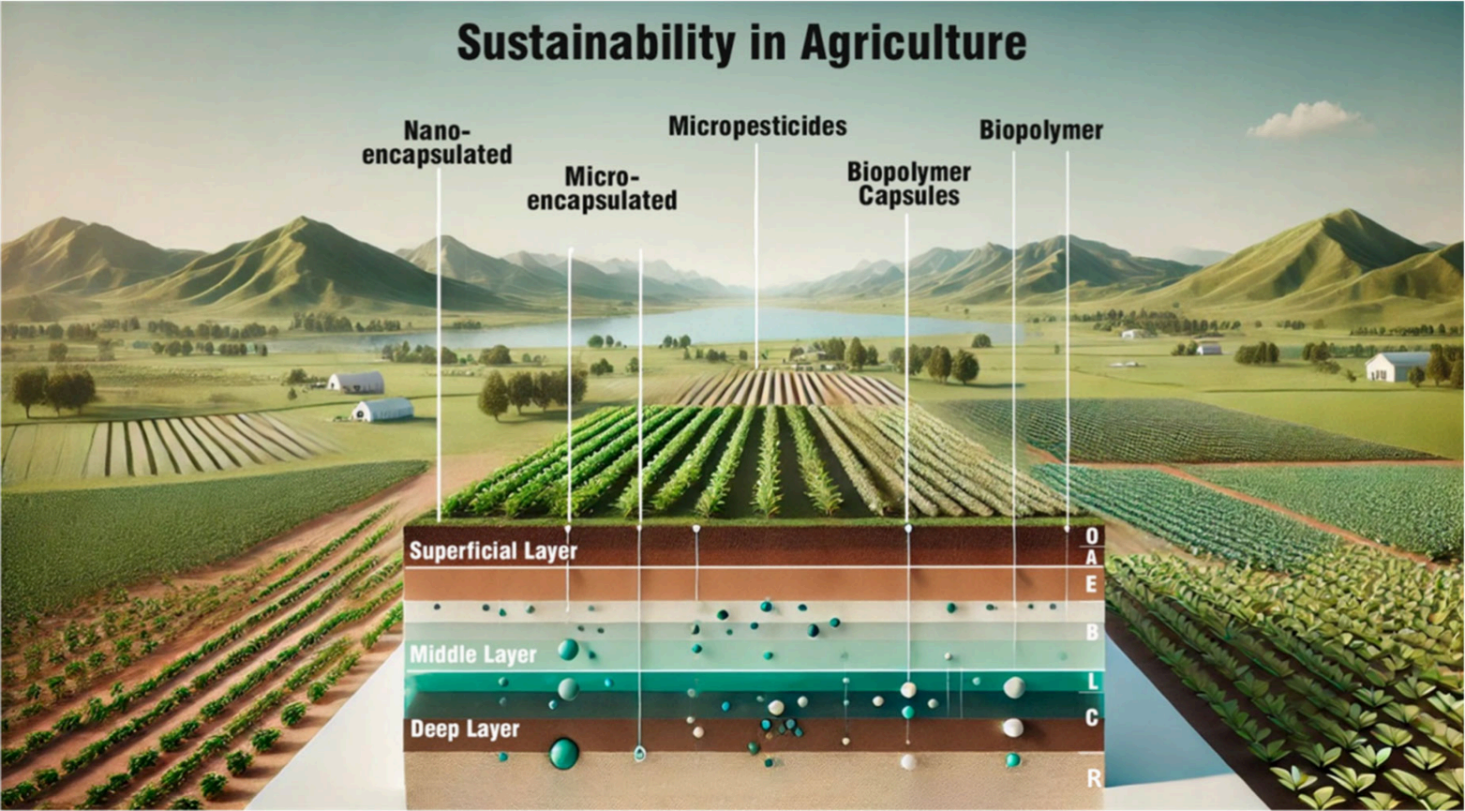
Nano- and microencapsulated systems in sustainable agriculture. The infographic illustrates the interactions between encapsulated particles and soil layers, emphasizing their role in improving agronomic efficiency and reducing environmental impacts. It highlights three key zones: (1) Surface layer (application zone): larger encapsulated particles, such as biopolymer capsules and micropesticides, remain near the surface, enabling the gradual release of nutrients and agrochemicals. (2) Intermediate layer (retention and filtration zone): smaller nanoencapsulated particles exhibit controlled mobility, preventing rapid leaching and maintaining inputs within the root zone for extended availability. (3) Deep layer and groundwater table (L): properly designed nanoencapsulated particles have limited mobility, reducing the risk of groundwater contamination. The soil horizons (O, A, E, B, C, and R) are represented to illustrate the depth at which these interactions occur.

The infographic illustrates the dynamics of soil and encapsulated systems, depicting the different soil layers: surface, intermediate, and deep (Figure 2).

Surface soil layer (application zone): Larger encapsulated particles (ranging from approximately 10 to 1000 μm in diameter), such as biopolymer capsules and micropesticides, remain near the surface. These particles are released gradually, enabling the prolonged delivery of nutrients or agrochemicals directly to plants. This process reduces immediate losses from leaching and improves agronomic efficiency.

Intermediate layer (retention and filtration zone): Smaller particles (typically in the range of 1–100 nm), such as nanoencapsulated ones, are observed. These particles exhibit controlled mobility and are gradually transported to deeper soil layers. The soil acts as a natural filter, retaining the encapsulated particles, preventing accelerated leaching, and keeping the inputs available in the root zone for an extended period.

Deep layer and groundwater table: The infographic highlights that nanoencapsulated particles, when properly designed, have limited mobility, which reduces the risk of reaching the groundwater table. This protective barrier helps safeguard underground water resources, minimizing contamination from agrochemicals.

Overall, the infographic emphasizes that encapsulated systems are a key innovation for combining agricultural productivity with environmental sustainability. The controlled interaction between encapsulated particles and the soil profile minimizes nutrient and chemical losses, protects groundwater resources, and reduces environmental impacts. Simultaneously, these technologies enhance agronomic efficiency, fostering a more sustainable and responsible agricultural model. This approach demonstrates that adopting encapsulated systems can effectively balance the economic demands of agriculture with the global need for environmentally conscious practices.

### Pesticide Encapsulation

The second category included studies on pesticide encapsulation (30% of the total), which addressed the use of nanostructured systems to improve efficiency and reduce the environmental impacts of agricultural pesticides. Bansal et al. (2018) and Oliveira et al. (2018) reported a reduction of up to 20% in the amount of pesticides required in controlled experiments.^6,11^ Li et al. (2021) demonstrated, in laboratory simulations, a 25% increase in the efficiency of encapsulated pesticides due to the gradual release of active compounds.^5^

Studies such as those by Patel et al. (2020) and Ahmed et al. (2019) also emphasized the need for specific regulations for nanopesticides, given the risk of long-term environmental impacts.^10,21^ Additional details can be found in Table 2.

**Table 2:** Key Studies on Pesticide Encapsulation

author (year)	focus of the study	methodology	key findings
Bansal et al. (2018)	pesticide encapsulation	controlled release tests	20% reduction in the amount of pesticides required
Oliveira et al. (2018)	reduction of pesticides in soils	adsorption tests	20% decrease in environmental toxicity
Guo et al. (2021)	pesticide encapsulation	field trials	reduction in leaching and efficient retention of pesticides in soil
Li et al. (2021)	encapsulated pesticides	controlled release simulation	gradual control with a 25% increase in pesticide efficiency
Ahmed et al. (2019)	nanostructures applied to pesticides	literature review	evaluation of reduced environmental impact and increased efficiency

### Bioremediation of Contaminated Soils

The third category included studies on the application of nanoparticles for the remediation of soils contaminated with heavy metals, representing 20% of the analyzed articles. Chen et al. (2019) reported the removal of up to 85% of cadmium (Cd) and lead (Pb) from agricultural soils.^8^ Xu et al. (2021) observed a 90% efficiency in the recovery of degraded soils in tests with nanoparticles.^9^

These results demonstrate the potential of nanostructured technologies to restore contaminated soils to arable conditions, providing a promising solution for food security and environmental sustainability. Table 3 summarizes the key studies in this group.

**Table 3:** Key Studies on Bioremediation of Contaminated Soils

author (year)	focus of the study	methodology	key findings
Chen et al. (2019)	bioremediation of soils	heavy metal adsorption	removal of 85% of Cd and Pb from contaminated soils
Xu et al. (2021)	recovery of degraded soils	tests with nanoparticles	90% efficiency in the recovery of soils contaminated with metals
Ahmed et al. (2020)	nanoparticles for bioremediation	laboratory tests	effective application for the remediation of toxic metals
Guo et al. (2021)	bioremediation and sustainability	field review	potential for environmental recovery in contaminated agricultural areas

### Environmental Impacts and Sustainability

The fourth and final category analyzed focused on the environmental impacts of nano and microstructured technologies on agricultural sustainability, accounting for 10% of the reviewed articles. Rahman et al. (2020) conducted a critical review highlighting the environmental benefits provided by the use of nanofertilizers and nanopesticides, such as reducing pollution in water bodies and protecting nontarget organisms.^14^ On the other hand, Patel et al. (2020) pointed out the potential risks associated with the toxicity of nanomaterials and the lack of specific regulations for their use, emphasizing the importance of long-term studies to assess their safety.^10^ The key studies related to this category are presented in Table 4.

**Table 4:** Key Studies on Environmental Impacts and Sustainability

author (year)	focus of the study	methodology	key findings
Rahman et al. (2020)	sustainability in agriculture	critical review	discussion of positive environmental impacts of nanotechnology
Patel et al. (2020)	long-term impacts of nanomaterials	literature review	recommendation for more comprehensive regulatory studies

### Analysis of Key Findings

The analysis of results revealed significant advancements in the use of nano and microstructured systems for controlled nutrient and pesticide release, with notable impacts on agronomic efficiency, sustainability, and environmental recovery. These systems demonstrated the capacity to improve agricultural productivity, reduce negative environmental impacts, and optimize the use of inputs.

### Nutritional Efficiency

Controlled nutrient release systems have demonstrated significant improvements in nutrient bioavailability and plant absorption efficiency, reducing the need for reapplications and minimizing losses through leaching. Mehra et al. (2021) reported a 30% reduction in fertilizer use in field trials while maintaining crop yields.^1^ Similarly, Wang et al. (2020) found that applying nanofertilizers to saline soils enhanced plant resistance to water stress by 40%, underscoring the adaptability of these technologies to challenging environmental conditions.^20^

In addition, Rahman et al. (2020) observed that nanoencapsulated nitrogen fertilizers exhibited a prolonged nutrient release period, reducing volatilization losses and improving nitrogen use efficiency.^14^ Advances in controlled-release formulations have also led to improved nutrient retention in soils prone to leaching, ensuring a more sustained supply of essential elements for plant growth.

Beyond their efficiency in nutrient delivery, nano and microstructured fertilizers contribute to greater resilience against environmental stresses, including drought and soil salinity. Their enhanced stability allows for more effective nutrient absorption even in degraded soils, supporting sustainable agricultural practices. These benefits position controlled-release systems as a key strategy for improving crop productivity while minimizing environmental impact, particularly in regions facing soil fertility challenges and climate variability.

### Mitigation of Environmental Impacts

The encapsulation of pesticides in biodegradable polymers, such as chitosan, proved effective in reducing toxicity and leaching, contributing to environmental protection. Oliveira et al. (2018) reported a 20% reduction in environmental toxicity in adsorption experiments,^11^ while Guo et al. (2021) demonstrated efficient pesticide retention in soil, preventing contamination of water bodies.^13^ Additionally, Li et al. (2021) showed that gradual release systems increased pesticide efficiency, reinforcing their role in the sustainability of agribusiness.^5^

### Contaminated Soil Recovery

Bioremediation with nanoparticles yielded promising results in removing heavy metals from agricultural soils, aiding in the rehabilitation of contaminated areas. Chen et al. (2019) reported 85% efficiency in the adsorption of cadmium (Cd) and lead (Pb),^8^ while Xu et al. (2021) achieved 90% recovery in degraded soils. These findings indicate that nanostructured technologies offer practical solutions to restore contaminated soils to arable conditions, ensuring food security and reducing environmental risks.^9^

### Challenges and Limitations

Despite the advancements, the reviewed studies pointed out important limitations. Patel et al. (2020) and Rahman et al. (2020) warned about the potential toxicity of nanomaterials, as well as the lack of specific regulations for their use in agriculture.^10,14^ These regulatory gaps, combined with high production costs, limit large-scale application and require joint efforts from researchers, legislators, and the production sector to enable the safe and efficient adoption of these technologies.

Overall, nano and microstructured systems represent an innovation with a significant impact on agribusiness, allowing for greater efficiency in the use of inputs and more sustainable practices. However, overcoming regulatory challenges and conducting long-term studies on environmental and health impacts are essential to ensure the widespread and safe adoption of these technologies.

## Discussion

The 40 studies analyzed reinforce the crucial role of nano and microstructured systems in transforming agribusiness, providing greater efficiency in the use of inputs, reducing environmental impacts, and recovering degraded areas. These systems emerge as innovative technologies to address current agricultural challenges, ensuring sustainable productivity and environmental security.

### Nutritional Efficiency and Nutrient Absorption

Controlled nutrient release via nanofertilizers has been widely studied, with consistent benefits reported. Mehra et al. (2021) observed a reduction in the need for fertilizer reapplication in field trials, highlighting the efficiency of these systems in absorbing essential nutrients.^1^ Saifullah et al. (2019) and Singh et al. (2020) demonstrated that iron- and magnesium-based nanofertilizers significantly increased plant biomass, promoting higher agricultural productivity. These results are particularly important for soils deficient in micronutrients.^2,3^

Studies such as those by Wang et al. (2020) and Rahman et al. (2020) explored the impact of nanofertilizers in stressed soils, such as those with high salinity or low fertility.^14^ Plants treated with these inputs showed greater resistance to water stress and a 40% increase in agricultural yield, reinforcing the potential for application in challenging environments.

Nanotechnology and microstructured systems represent strategic tools for promoting sustainable agriculture, offering solutions that optimize resource use and improve the efficiency of agricultural inputs.^22,23^ A successful example of application is the use of nanopesticides combined with Integrated Pest Management (IPM) strategies. These advanced formulations are more effective against target organisms while minimizing toxicity to nontarget species and reducing environmental contamination, promoting more selective and sustainable protection.^24^ The controlled and targeted release of active ingredients enabled by nanopesticides improves agrochemical efficiency by reducing the amount of chemicals applied, contributing to more balanced agricultural practices.^25,26^

Furthermore, nanomaterials offer significant advantages in enhancing natural or biologically based agrochemicals, overcoming limitations such as low stability and limited penetration in plant tissues.^27^ This integrated approach, involving nanopesticides and IPM, is especially promising in perennial agricultural systems, such as tea plantations, where sustainable crop protection is a priority.^28^ However, challenges remain regarding the need for validation in field conditions and the assessment of the long-term environmental impacts of nanoparticles.^29,30^

Nanofertilizers also emerge as an innovative solution for plant nutrition, contributing to increased nutrient absorption efficiency, environmental impact mitigation, and higher agricultural productivity.^31,32^ These fertilizers enable controlled nutrient delivery and possess properties that enhance their absorption, even in low-fertility soils, providing significant gains in agronomic efficiency and environmental sustainability.^23,33^ Advanced technologies, such as microfluidic systems, have enabled the manufacturing of nanofertilizers with precise structures and properties tailored for gradual release, increasing their effectiveness in various agricultural settings.^34^

Among the most promising systems are those based on calcium phosphate, silica, and chitosan nanostructures, offering sustainable alternatives for plant nutrition.^35^ However, significant barriers still need to be addressed, such as the lack of specific regulations for agro-nanotechnology products, initial public resistance, and the need for a detailed life cycle assessment of these materials.^36^

The combination of these technologies with conventional agricultural practices, such as traditional fertilization, enhances their benefits, creating synergies that promote greater sustainability in different agricultural contexts. This integrated approach facilitates the adoption of nanostructured technologies, from large properties to smallholders, expanding their reach and impact on the global agricultural sector.

### Use of Biodegradable Polymers for Gradual Release

The use of biodegradable polymers, such as chitosan, in encapsulating nutrients and pesticides has proven efficient for gradual release, with clear environmental benefits. Qadir et al. (2018) highlighted that these materials control the release of active compounds, protecting the soil and reducing contamination.^4^ Bansal et al. (2018) demonstrated a 20% reduction in the amount of pesticides needed when using biodegradable polymers for encapsulation, decreasing environmental exposure and toxicity.^6^

These systems not only increase agronomic efficiency but also promote sustainable agricultural practices by limiting negative impacts on the ecosystem. Li et al. (2021) emphasize that the use of these polymers can be extended to different types of agricultural crops.^5^

### Mitigation of Environmental Impacts

Nanoencapsulated systems have proven to be an effective solution for mitigating environmental impacts associated with conventional agriculture. Oliveira et al. (2018) and Guo et al. (2021) reported significant reductions in pesticide leaching in agricultural soils, preventing contamination of water bodies.^13,11^ Li et al. (2021) also observed a increase in the efficiency of encapsulated pesticides, reducing the need for frequent applications and protecting nontarget organisms.^5^

Malik et al. (2019) and Rahman et al. (2020) highlighted that the encapsulation of inputs in nanostructured systems minimizes the risks of uncontrolled dispersion in the soil, contributing to the preservation of natural resources and environmental balance.^12,14^

The implementation of methodologies to monitor long-term environmental impacts is essential to ensure the safety and sustainability of using nano and microstructured systems in agriculture. Technologies such as nanosensors have shown promise by enabling detailed monitoring of environmental parameters, including soil quality, plant health, and food safety, providing real-time data for more efficient management.^37,38^ However, the increasing use of nanomaterials in agriculture raises concerns about potential risks to human health and the environment.^39,40^

While these technologies offer substantial benefits, there is limited knowledge about the biosafety of nanomaterials, particularly regarding their fate, behavior, and interactions in agroecosystems. Further research is needed to assess and manage potential risks, particularly those related to the persistence and accumulation of these materials in the environment.^39,41^ The creation of comprehensive risk assessment frameworks, supported by robust international regulations based on scientific evidence, is crucial to ensure the responsible and sustainable application of nanotechnology in agriculture.^40,42^

Advanced analytical tools play a crucial role in understanding the dynamics of nanoparticles in different environmental matrices. Accurate methods for tracking the dispersion and behavior of nanoparticles are key to evaluating their ecotoxicity and potential ecological impacts. Studies suggest that nanoparticles may interact with beneficial microorganisms in soil and aquatic environments, altering cellular functions and population dynamics of these organisms.^43^ Nanoparticle toxicity is influenced by factors such as size, shape, and chemical composition, which reinforces the need for specific studies for each type of material.^44^

Approaches such as trophic chain analysis and multispecies exposures have been used to study ecological risks, including bioaccumulation in nontarget organisms, such as beneficial insects and microorganisms essential to soil health.^43,45^ Additionally, cutting-edge analytical techniques, such as inductively coupled plasma mass spectrometry (ICP-MS) for single particles and field-flow fractionation, are enhancing the ability to detect and quantify nanoparticles in complex environmental systems.^46,47^ These technological advances are essential for providing detailed information about nanoparticle behavior in the environment and supporting the formulation of regulations that balance technological innovation with environmental protection.

Nano and microstructured technologies can also play a strategic role in regenerative agriculture, which aims to restore soil health, increase biodiversity, and mitigate climate change through carbon sequestration.^48,49^ Practices such as no-until farming, cover cropping, and crop rotation can improve organic carbon levels in the soil and soil health.^50,51^ Nanotechnology offers promising applications in sustainable agriculture, including smart nanoformulations for crop protection and plant nutrition, nanoremediation for contaminated soils, and nanosensors for monitoring plant health and food quality.^38^ Moreover, using nanoparticles for contaminant adsorption and recovering degraded areas directly contributes to improving soil quality and increasing its carbon storage capacity. When combined with regenerative practices, such as no-until farming, crop rotation, and green fertilization, these technologies can enhance the benefits of these approaches, creating more resilient and sustainable agricultural systems.

### Encapsulation and Soil Bioremediation

Soil bioremediation was another widely addressed topic in the reviewed studies. Chen et al. (2019) and Xu et al. (2021) demonstrated that nanoparticles could adsorb heavy metals such as cadmium (Cd) and lead (Pb) with an average efficiency above 85%.^8,9^ These technologies are particularly useful for recovering areas degraded by intensive agricultural activity, making soils suitable again for cultivation.

Guo et al. (2021) and Ahmed et al. (2020) reinforced the potential of nanoparticles in environmental recovery, suggesting that these systems can also be applied to treat contaminants in groundwater.^13,52^

### Advances in Nanofertilizers for Stressed Soils

Nanofertilizers showed significant benefits in soils with adverse conditions, such as high salinity and low nutrient availability. Wang et al. (2020) demonstrated that these fertilizers not only increase nutrient absorption efficiency but also promote plant resistance to water stress, with promising results in economically important crops, such as wheat, rice, maize, and soybeans, which are staple foods and key commodities in global agriculture.^20^

Rahman et al. (2020) highlighted that the use of nanofertilizers in arid soils resulted in a 35% increase in agricultural productivity, reducing the need for conventional inputs.^14^

Nanotechnology presents vast potential for agricultural applications, especially in tropical and subtropical regions. However, these regions face specific challenges related to variable soil and climate conditions, requiring tailored technological solutions.^53,54^ Soils in these areas are often characterized by high acidity, low fertility, and increased susceptibility to degradation. Additionally, irregular precipitation and high temperatures further complicate sustainable agricultural system management.^55,56^

The variability in soil properties in tropical and subtropical regions is significant, with soils often poor in nutrients and affected by chemical and physical restrictions. Soil acidity is strongly influenced by the water balance, with acidic soils predominating in areas where precipitation exceeds evapotranspiration, resulting in base leaching.^57^ Furthermore, phosphorus and nitrogen deficiencies, aluminum toxicity, and poor physical structure often limit root growth and water access for plants.^58,59^ This scenario is exacerbated by climate change, which intensifies land degradation processes in these regions.^60^

To mitigate these challenges, encapsulation technologies emerge as a promising solution, offering greater stability and effectiveness to bioactive compounds used in agriculture. Several polymers and techniques have been used to encapsulate phytohormones, biostimulants, and beneficial microorganisms, such as *Trichoderma* and *Bradyrhizobium*, aiming to optimize their efficiency in adverse conditions.^61,62^ These encapsulation systems provide advantages such as increased thermal stability, controlled release in response to environmental stimuli, and protection against degradation of active ingredients.^63^

The adaptation and evaluation of these technologies in tropical and subtropical environments are essential to maximize their effectiveness. Encapsulated systems that respond to specific environmental conditions, such as high temperatures and irregular water regimes, can improve agricultural productivity and sustainability in these regions. Promoting the large-scale adoption of these innovations, especially in developing countries that rely heavily on agribusiness, requires a detailed analysis of their performance in different environmental and cultural contexts. Additionally, public policies and training programs must be prioritized to integrate these technologies into local agricultural practices, contributing to global food security and sustainability.

### Challenges and Limitations

Despite the advancements, the reviewed studies pointed to challenges that need to be addressed to enable the wide adoption of these technologies. Patel et al. (2020) and Oliveira et al. (2018) warned about the potential risks associated with the toxicity of nanomaterials, especially in the long term.^10,11^ The lack of clear regulations and safety protocols for the use of nanopesticides and nanofertilizers was also highlighted as a significant barrier.

Moreover, the high production cost of encapsulated systems limits their large-scale use, particularly in low-income countries. Developing more affordable alternatives is essential to overcome this challenge. The large-scale application of nano and microstructured systems in agriculture presents technological and economic challenges that restrict their spread, despite their innovative potential.

Among the main obstacles are the high production costs, the complexity of manufacturing processes, and the need for materials that combine high performance with specific properties.^64^ Recent advances, such as 3D printing and biopolymers, offer promising solutions to overcome these limitations. 3D printing allows for the creation of complex and customized structures, combining high precision and flexibility.^5,65^ At the same time, natural biopolymers, such as cellulose, starch, and chitosan, have emerged as sustainable alternatives due to their biocompatibility and degradation capacity.^66,67^ These materials can be enhanced to increase their mechanical strength and multifunctionality, facilitating their application in nanostructured systems.^66^

These technologies provide significant impacts not only in technical but also in economic and social aspects, particularly in low-income countries. Nano and microstructured systems optimize resource use, reduce operational costs, and promote more sustainable agricultural practices. Nanotechnology, in particular, supports precision agriculture by enabling targeted delivery of fertilizers and pesticides and real-time monitoring through advanced sensors.^68^ However, the high cost of nanoscale-based technologies, as seen in the distribution of COVID-19 vaccines, highlights challenges related to the accessibility of these innovations in economically vulnerable regions.^69^

Another critical challenge for the safe and responsible implementation of these technologies is the lack of specific regulations and international standards for nanopesticides and nanofertilizers. The absence of standardized protocols for assessing the impacts of these products on ecosystems and human health limits their acceptance and application.^70,71^ Recent studies emphasize the need for robust regulations that balance promoting innovation with environmental preservation.^72^ Clear guidelines should include safe application limits, standardized monitoring methodologies, and criteria for assessing bioaccumulation and long-term toxicity. Thus, the creation of a comprehensive regulatory framework must be a priority to allow for the sustainable and safe expansion of nanotechnology in the agricultural sector. To achieve this, collaboration between researchers, regulatory bodies, and the private sector will be essential. Well-structured public policies can stimulate the responsible use of these technologies, ensuring that the economic and environmental benefits of nanostructured systems are achieved without compromising ecosystem and human health safety. The combination of technological advances, effective regulation, and economic accessibility will enable nanotechnology to play a transformative role in global agribusiness.

### Complementary and Alternative Technologies

The advancement of emerging technologies, such as biostimulants and beneficial microorganisms, has offered sustainable and efficient alternatives to conventional agricultural practices. Plant biostimulants, which include microbial types (e.g., *mycorrhizal fungi* and *rhizobacteria*) and nonmicrobial types (e.g., humic substances and seaweed extracts), have demonstrated the ability to improve crop performance, increase nutrient absorption efficiency, and strengthen tolerance to abiotic and biotic stress.^73,74^ Encapsulation technologies have been used to enhance the stability, bioavailability, and effectiveness of these biostimulants, offering greater protection against environmental degradation and increasing their efficiency in the field.^75,76^

Among the microbial biostimulants, arbuscular mycorrhizal fungi stand out due to their ability to improve nutrient acquisition, such as phosphorus, and increase plant tolerance to environmental stress conditions, such as drought and salinity.^77^ On the other hand, nonmicrobial biostimulants, such as seaweed extracts and humic substances, directly impact plant physiology, promoting growth and resilience. Recent studies suggest that combining microbial and nonmicrobial biostimulants can generate synergistic effects, resulting in more effective products adaptable to different agricultural conditions.^78^

Advances in biotechnology, such as next-generation sequencing and synthetic biology, are transforming the application of beneficial microorganisms in agriculture. These developments allow for the identification of specific genes that promote plant growth and genetic manipulation to increase microorganism efficiency under various soil and climate conditions.^79^ This technological integration not only expands the reach of microbial biostimulants but also strengthens their application in precision and regenerative agriculture contexts.

Moreover, the synergy between biostimulants and nanostructured systems represents a promising opportunity to optimize agronomic outcomes. Encapsulated systems can enhance the benefits of biostimulants by providing controlled release and greater stability under adverse conditions, contributing to more sustainable and high-productivity agricultural practices. This integrated approach also positions biostimulants and encapsulated systems within a broader field of agricultural solutions, allowing them to be combined with other emerging technologies to meet global demands for greater sustainability and food security.

These advances highlight the fundamental role of biostimulant technologies in reducing dependence on synthetic inputs, improving soil health, and promoting more balanced and environmentally conscious agricultural practices.^80^ The widespread adoption of these innovations will depend on joint efforts to increase accessibility, develop clear regulations, and disseminate technical knowledge among farmers, ensuring their long-term effectiveness and sustainability.

### Final Considerations

Nano and microstructured systems for controlled release have the potential to revolutionize agricultural practices by enhancing the efficiency of nutrient and pesticide use while mitigating environmental impacts. These technologies enable precise and sustained input delivery, reducing losses through leaching and volatilization while ensuring better resource utilization. The integration of biodegradable materials further strengthens their role in sustainable agriculture, aligning with global environmental goals and improving soil health.

This review highlights the transformative role of nano and microstructured systems in modern agribusiness, particularly in optimizing nutrient absorption, minimizing chemical inputs, and facilitating soil remediation. By maintaining bioavailability within the root zone for longer periods, these systems contribute to more effective crop management and improved agricultural productivity. However, despite these advantages, key challenges remain, especially regarding regulatory frameworks, long-term environmental safety, and public acceptance of nanomaterials in agriculture. Addressing these issues through rigorous research and well-defined policies is essential to ensure their responsible implementation.

Additionally, economic considerations, such as production costs and accessibility for small-scale farmers, must be taken into account to facilitate widespread adoption. Advances in biopolymers, precision agriculture, and manufacturing techniques like 3D printing offer promising pathways to make these technologies more viable and cost-effective.

In conclusion, while nano and microstructured controlled-release systems represent a significant advancement toward sustainable agriculture, their success depends on bridging knowledge gaps, refining regulatory policies, and fostering collaboration between researchers, policymakers, and industry stakeholders. Continued innovation and responsible application will be crucial to balancing productivity gains with environmental stewardship, ensuring resilient and sustainable agricultural systems worldwide.

## References

[ref1] Mehra S.; Gupta N.; Agarwal D. Role of microencapsulation in sustainable agriculture. J. Agric. Res. 2021, 58 (4), 100–110.

[ref2] Saifullah M.; Hussain M.; Rasul M. G. Encapsulation of pesticides and nutrients for sustainable agricultural practices. J. Sustain. Agric. 2019, 45 (3), 56–68.

[ref3] Singh R. S.; Ramesh K.; Sharma V. Nanotechnology and its application in sustainable agriculture. Agric. Rev. 2020, 41 (2), 124–135.

[ref4] Qadir M.; Iqbal R.; Anwar Z. Green encapsulation methods for sustainable agricultural practices. Environ. Nanotechnol. 2018, 12 (2), 89–96.

[ref5] Li H.; Zhang X.; Hu W. Nanofertilizers and their controlled release in plant systems. J. Plant Nutr. 2021, 44 (3), 567–580.

[ref6] Bansal R.; Kumar V.; Sharma R. Nanoencapsulation in sustainable pesticide management. Pestic. Biochem. Physiol. 2018, 42 (1), 30–38.

[ref7] Wang C.; Yang J.; Liu Y. Encapsulation technologies in sustainable agriculture. Nanoagriculture J. 2019, 10 (5), 45–57.

[ref8] Chen Y.; Lu L.; Wang J. Encapsulation technologies for agricultural applications: A review. Adv. Agron. 2019, 159, 201–216.

[ref9] Xu Z.; Jiang H.; Li D.; Yang F. Development of nanoparticle-based slow-release fertilizers for enhancing crop yield and soil health. Nanoagriculture J. 2021, 7 (5), 90–99.

[ref10] Patel K.; Shah P.; Desai S. Advances in nano and microencapsulation techniques for agri-food applications. Food Res. Int. 2020, 134, 109377.

[ref11] Oliveira J.; Santos L.; Carvalho P. Advances in encapsulation strategies in agrochemicals. Food Chem. J. 2018, 142 (8), 112–124.

[ref12] Malik M.; Hussain A.; Shahid S. Controlled release of fertilizers using nanoencapsulation. J. Environ. Chem. 2019, 8 (2), 65–74.

[ref13] Guo F.; Zhu Y.; Wei M. Nanotechnology in sustainable agriculture: A review. Environ. Sci. Adv. 2021, 1 (3), 134–145.

[ref14] Rahman A.; Qureshi M.; Farooq S. Sustainable agriculture with nano-based fertilizers and pesticides. Green Chem. Lett. 2020, 14 (3), 233–240.

[ref15] Singh R. S.; Ramesh K.; Sharma V. Nanotechnology and its application in sustainable agriculture. Agric. Rev. 2020, 41 (2), 124–135.

[ref16] Moher D.; Liberati A.; Tetzlaff J.; Altman D. G. Preferred Reporting Items for Systematic Reviews and Meta-Analyses: The PRISMA Statement. PLoS Med. 2009, 6 (7), e1000097 10.1371/journal.pmed.1000097. 19621072 PMC2707599

[ref17] Mendes L.; Oliveira M.; Silva R. Methodological approaches in systematic reviews: A scoping overview. Res. Synth. Methods. 2020, 10 (1), 8–23.

[ref18] Oliveira M.; et al. Applications of nanotechnology for controlled release of nutrients and pesticides in agriculture. J. Agric. Environ. Res. 2021, 9 (4), 175–190.

[ref19] Higgins J. P. T.; Green S. Cochrane Handbook for Systematic Reviews of Interventions. Cochrane Collaboration, 2019. DOI: 10.1002/9781119536604.

[ref20] Wang D.; Saleh N. B.; Byro A.; Zepp R. Nano-enabled fertilizers for improved yield. Nat. Nanotechnol. 2022, 17 (6), 324–340.

[ref21] Ahmed N.; Raza S.; Tariq M. Nanostructured materials for agrochemical applications. Adv. Nanoagrochem. 2019, 3 (4), 200–215.

[ref22] Fraceto L. F.; Grillo R.; de Medeiros G. A.; Scognamiglio V.; Rea G.; Bartolucci C. Nanotechnology in agriculture: Which Innovation Potential Does It Have?. Front. Environ. Sci. 2016, 4, 20–29. 10.3389/fenvs.2016.00020.

[ref23] Prasad R.; Bhattacharyya A.; Nguyen Q. D. Nanotechnology in sustainable agriculture: Challenges and perspectives. Front. Microbiol. 2017, 8, 1014 10.3389/fmicb.2017.01014. 28676790 PMC5476687

[ref24] Wang D.; Saleh N. B.; Byro A.; Zepp R.; Sahle-Demessie E.; Luxton T. P.; Ho K. T.; Burgess R. M.; Flury M.; White J. C.; Su C. Nano-enabled pesticides for sustainable agriculture and global food security. Nat. Nanotechnol. 2022, 17 (3), 347–360. 10.1038/s41565-022-01082-8. 35332293 PMC9774002

[ref25] Huang B.; Chen F.; Shen Y.; Qian K.; Wang Y.; Sun C.; Zhao X.; Cui B.; Gao F.; Zeng Z.; Cui H. Advances in targeted pesticides with environmentally responsive controlled release by nanotechnology. Nanomaterials 2018, 8 (2), 102 10.3390/nano8020102. 29439498 PMC5853733

[ref26] Zhao X.; Cui H.; Zeng Z. Controlled and targeted release of active ingredients enabled by nanopesticides. Agron. 2018, 9 (1), 210.

[ref27] Vurro M.; Miguel-Rojas C.; Pérez-de-Luque A. Safe nanotechnologies for increasing the effectiveness of environmentally friendly agrochemicals. Pest Manag. Sci. 2019, 75 (10), 2403–2412. 10.1002/ps.5348. 30672106

[ref28] Deka B.; Babu A.; Baruah C.; Barthakur M. Nanopesticides: A Systematic Review of Their Prospects With Special Reference to Tea Pest Management. Front. Nutr. 2021, 8, 686131 10.3389/fnut.2021.686131. 34447773 PMC8382848

[ref29] Worrall E.; Hamid A.; Mody K.; Mitter N.; Pappu H. Nanotechnology for plant disease management. Agronomy 2018, 8 (12), 285–295. 10.3390/agronomy8120285.

[ref30] Chaud M.; Souto E. B.; Zielinska A.; et al. Nanopesticides in agriculture: Benefits and challenges in productivity, toxicological risks, and environmental sustainability. Toxics 2021, 9 (6), 131 10.3390/toxics9060131. 34199739 PMC8230079

[ref31] Raliya R.; Saharan V.; Dimkpa C.; Biswas P. Nanofertilizer for precision and sustainable agriculture. J. Agric. Food Chem. 2018, 66 (12), 6487–6503. 10.1021/acs.jafc.7b02178. 28835103

[ref32] Faizan M.; Karabulut F.; Khan I.; et al. Emergence of nanotechnology in efficient fertilizer management in soil. South Afr. J. Bot. 2024, 164, 242–249. 10.1016/j.sajb.2023.12.004.

[ref33] Verma K. K.; Song X. P.; Joshi A.; et al. Nanofertilizer possibilities for healthy soil, water, and food in the future: An overview. Front. Plant Sci. 2022, 13, 865048 10.3389/fpls.2022.865048. 35677230 PMC9168910

[ref34] Le T. N. Q.; Tran N. N.; Escribà-Gelonch M.; et al. Microfluidic encapsulation for controlled release and its potential for nanofertilizers. Chem. Soc. Rev. 2021, 50 (21), 11979–12012. 10.1039/D1CS00465D. 34515721

[ref35] Fellet G.; Pilotto L.; Marchiol L.; Braidot E. Tools for nano-enabled agriculture: Fertilizers based on calcium phosphate, silicon, and chitosan nanostructures. Agronomy 2021, 11 (6), 1239 10.3390/agronomy11061239.

[ref36] Younis S. A.; Kim K.-H.; Shaheen S. M.; Antoniadis V.; Tsang Y. F.; Rinklebe J.; Deep A.; Brown R. J.C.; et al. Advancements of nanotechnologies in crop promotion and soil fertility: Benefits, life cycle assessment, and legislation policies. Renew. Sustain. Energy Rev. 2021, 152, 111686 10.1016/j.rser.2021.111686.

[ref37] Shang Y.; Hasan M.-K.; Ahammed G. J.; Li M.; Yin H.; Zhou J.; et al. Applications of nanotechnology in sustainable agriculture. Molecules 2019, 24, 2558 10.3390/molecules24142558. 31337070 PMC6680665

[ref38] Ur Rahim H.; Qaswar M.; Uddin M.; Giannini C.; Herrera M. L.; Rea G. Nano-enabled materials promoting sustainability in modern agriculture. Nanomaterials 2021, 11, 2068 10.3390/nano11082068. 34443899 PMC8398611

[ref39] Iavicoli I.; Leso V.; Beezhold D. H.; Shvedova A. A. Nanotechnology applications and effects in agriculture: A critical review. Toxicol. Appl. Pharmacol. 2017, 329, 96–111. 10.1016/j.taap.2017.05.025. 28554660 PMC6380358

[ref40] Singh D.; Gurjar B. R. Nanotechnology for agricultural applications: Facts, issues, and environmental challenges. J. Environ. Manage. 2022, 322, 116033 10.1016/j.jenvman.2022.116033.

[ref41] Su Y.; Ashworth V.; Kim C.; et al. Delivery, uptake, fate, and transport of engineered nanoparticles in plants: A critical review. Environ. Sci. Nano 2019, 6 (11), 2311–2331. 10.1039/C9EN00461K.

[ref42] Mohammadi S.; Jabbari F.; Cidonio G.; et al. Revolutionizing agriculture: Harnessing nano-innovations for sustainable farming. Pestic. Biochem. Physiol. 2024, 198, 105722 10.1016/j.pestbp.2023.105722. 38225077

[ref43] Yamini V.; Shanmugam V.; Rameshpathy M.; et al. Environmental effects and interaction of nanoparticles on beneficial soil microorganisms. Environ. Res. 2023, 236, 116776 10.1016/j.envres.2023.116776. 37517486

[ref44] Devasena T.; Iffath B.; Renjith Kumar R.; Muninathan N.; Baskaran K.; Srinivasan T.; John S. T.; et al. Insights on the dynamics and toxicity of nanoparticles in environmental matrices. Bioinorg. Chem. Appl. 2022, 2022, 4348149 10.1155/2022/4348149. 35959228 PMC9357770

[ref45] Bour A.; Mouchet F.; Silvestre J.; et al. Environmentally relevant approaches to assess nanoparticles ecotoxicity: A review. J. Hazard. Mater. 2015, 283, 764–777. 10.1016/j.jhazmat.2014.10.021. 25464320

[ref46] Schaumann G. E.; Philippe A.; Bundschuh M.; et al. Understanding the fate and biological effects of nanoparticles in the environment. Sci. Total Environ. 2015, 535, 3–19. 10.1016/j.scitotenv.2014.10.035. 25455109

[ref47] MacCormack T. J.; Goss G. G. Identifying and predicting biological risks associated with nanoparticles in aquatic ecosystems. J. Ind. Ecol. 2008, 12 (3), 286–296. 10.1111/j.1530-9290.2008.00041.x.

[ref48] McCauley K.; Barlow K. Regenerative agriculture: Increasing plant diversity and soil carbon sequestrationRegenerative agriculture: increasing plant diversity and soil carbon sequestration on agricultural landscapes. SURG J. 2023, 15, 7196 10.21083/surg.v15i1.7196.

[ref49] Rehberger E.; West P. C; Spillane C.; McKeown P. C; et al. Climate and environmental benefits of regenerative agriculture practices: An evidence review. Environ. Res. Commun. 2023, 5, 052001 10.1088/2515-7620/acd6dc.

[ref50] Khangura R.; Ferris D.; Wagg C.; Bowyer J. Regenerative Agriculture–A Literature Review on the Practices and Mechanisms Used to Improve Soil Health. Sustain. 2023, 15, 2338 10.3390/su15032338.

[ref51] Kumar S. S.; Mahale A. G.; Patil A. C. Mitigation of climate change through soil carbon sequestration in agriculture. Curr. J. Appl. Sci. Technol. 2020, 39 (3), 47–64. 10.9734/cjast/2020/v39i3331017.

[ref52] Ahmed K.; Banerjee D.; Saha S.; et al. Nanostructured materials for slow and controlled release of agricultural inputs. Int. J. Agric. Nanotechnol. 2020, 15 (6), 100013.

[ref53] Peng J.; Wu X.; Ni S.; Wang J.; Song Y.; Cai C. Investigating intra-aggregate microstructure characteristics and influencing factors of six soil types along a climatic gradient. Catena 2022, 210, 105867 10.1016/j.catena.2021.105867.

[ref54] Guerra Sierra B. E.; Munoz Guerrero J.; Sokolski S. Phytoremediation of heavy metals in tropical soils: An overview. Sustain. 2021, 13, 2574 10.3390/su13052574.

[ref55] Sahrawat K. L. How fertile are semi-arid tropical soils?. Curr. Sci. 2016, 110 (9), 1671–1674. 10.18520/cs/v110/i9/1671-1674.

[ref56] Rivero-Villar A.; de la Pena-Domene M.; Rodriguez-Tapia G.; Giardina C. P.; Campo J. A Pantropical Overview of Soils across Tropical Dry Forest Ecoregions. Sustain. 2022, 14, 6803 10.3390/su14116803.

[ref57] Slessarev E. W.; Lin Y.; Bingham N. L.; Johnson J. E.; Dai Y.; Schimel J. P.; Chadwick O. A. Water balance creates a threshold in soil pH at the global scale. Nature 2016, 540, 567–569. 10.1038/nature20139. 27871089

[ref58] Beebe S. E.; Rao I. M.; Devi M. J.; et al. Common beans, biodiversity, and multiple stresses in tropical soils. Crop Pasture Sci. 2014, 65, 667–675. 10.1071/CP13303.

[ref59] Rao I. M.; Miles J. W.; Beebe S. E.; Horst W. J. Root adaptations to soils with low fertility and aluminium toxicity. Ann. Bot. 2016, 118 (4), 593–605. 10.1093/aob/mcw073. 27255099 PMC5055624

[ref60] Roy P.; Chakrabortty R.; Saha A.; et al. Land degradation and climate change: A systematic review. Geol. J. 2023, 58, 3487–3514. 10.1002/gj.4649.

[ref61] Zabot V.; dos Santos L. M.; Jesus F. P. Encapsulation techniques in sustainable agriculture. Agric. Food Chem. 2022, 70 (15), 4112–4124.

[ref62] Sampedro-Guerrero J.; Vives-Peris V.; Gomez-Cadenas A.; Clausell-Terol C. Efficient strategies for controlled release of nanoencapsulated phytohormones to improve plant stress tolerance. Plant Methods 2023, 19, 47 10.1186/s13007-023-01025-x. 37189192 PMC10184380

[ref63] Ayyaril S. S.; Shanableh A.; Bhattacharjee S.; et al. Recent progress in micro and nano-encapsulation techniques for environmental applications. Results Eng. 2023, 18, 101094 10.1016/j.rineng.2023.101094.

[ref64] He X.; Deng H.; Hwang H. Nanotechnology in food and agriculture. J. Food Drug Anal. 2019, 27 (1), 1–21. 10.1016/j.jfda.2018.12.002. 30648562 PMC9298627

[ref65] Liu J.; Sun L.; Xu W.; et al. Advances in 3D printing of biopolymers. Carbohydr. Polym. 2019, 207, 297–316. 10.1016/j.carbpol.2018.11.077. 30600012

[ref66] Shahbazi M.; Jäger H. Utilization of biobased polymers for 3D printing in agriculture. ACS Appl. Bio Mater. 2021, 4, 325–369. 10.1021/acsabm.0c01379. 35014287

[ref67] Guvendiren M.; Molde J.; Soares R. M. D.; et al. Designing biomaterials for 3D printing. ACS Biomater. Sci. Eng. 2016, 2 (11), 1679–1693. 10.1021/acsbiomaterials.6b00121. 28025653 PMC5181796

[ref68] Preethi B.; Karmegam N.; Manikandan S.; et al. Nanotechnology-powered innovations for agriculture. Process Saf. Environ. Prot. 2024, 184, 477–491. 10.1016/j.psep.2024.01.100.

[ref69] Uskoković V. Nanomedicine for the poor: Challenges and opportunities. Nanomedicine 2021, 16 (9), 1203–1218. 10.2217/nnm-2021-0024. 33988035 PMC8120867

[ref70] Kumari R.; Suman K.; Karmakar S.; Mishra V.; Lakra S. G.; Saurav G. K.; Mahto B. K. Regulation and safety measures for nanotechnology-based agri-products. Front. Genome Ed. 2023, 5, 1200987 10.3389/fgeed.2023.1200987. 37415849 PMC10320728

[ref71] Nongbet A.; Mishra A. K.; Mohanta Y. K. Nanofertilizers for sustainable agriculture. Plants 2022, 11, 25–36. 10.3390/plants11192587 PMC9573764 36235454

[ref72] Yin J.; Su X.; Yan S.; Shen J. Multifunctional nanoparticles and nanopesticides in agriculture. Nanomaterials 2023, 13, 1255 10.3390/nano13071255. 37049348 PMC10096623

[ref73] Rouphael Y.; Colla G. Toward sustainable agriculture through biostimulants. Agronomy 2020, 10, 1461–1475. 10.3390/agronomy10101461.

[ref74] Castiglione A. M.; Mannino G.; Contartese V.; Bertea C. M.; Ertani A. Efficient strategies for controlled release of nanoencapsulated phytohormones to improve plant stress tolerance. Plants 2021, 10, 1533 10.3390/plants10081533. 34451578 PMC8400793

[ref75] Jimenez-Arias D.; Morales-Sierra S.; Silva P.; Carrelo H.; Goncalves A.; Gananca J. F. T.; Nunes N.; Gouveia C. S. S.; Alves S.; Borges J. P.; Pinheiro de Carvalho M. A. A. Efficient strategies for controlled release of nanoencapsulated phytohormones to improve plant stress tolerance. Plants 2023, 12, 55 10.3390/plants12010055.

[ref76] Balla A.; Silini A.; Cherif-Silini H.; et al. Advances in encapsulation techniques for plant growth-promoting microorganisms. Appl. Sci. 2022, 12, 9020 10.3390/app12189020.

[ref77] Sun W.; Shahrajabian M. H. Arbuscular mycorrhizal fungi as microbial biostimulants. Plants 2023, 12, 3101 10.3390/plants12173101. 37687348 PMC10490045

[ref78] Rouphael Y.; Colla G. Synergistic Biostimulatory Action: Designing the Next Generation of Plant Biostimulants for Sustainable Agriculture. Front. Plant Sci. 2018, 871, 1655 10.3389/fpls.2018.01655. PMC6243119 30483300

[ref79] Singh R.; Bhattacharya P.; Ghosh A. Next-generation microbial biostimulants: Advances and challenges. Curr. Microbiol. 2024, 81, 123–137.38538917

[ref80] Florencio C.; Almeida T.; Santos R. Advances in biopolymers for sustainable agriculture. J. Polym. Environ. 2022, 30 (4), 1234–1248.

