# Correction to “Nano- and Microstructured Systems for Controlled Release of Agricultural Inputs: Innovations for Efficiency and Sustainability”

**DOI:** 10.1021/acs.jafc.5c14145

**Published:** 2025-11-18

**Authors:** Aline Rombega Tito Rosa, Renato Farias do Valle Júnior, Marcos Vinicius da Silva, Hugo Felix Perini, Carlo José Freire Oliveira, Rodrigo César Rosa, Antonio Carlos Shimano, Anielle Christine Almeida Silva, Luís Carlos de Morais

In the original version of this Review Article, several references were incorrectly reported due to a malfunction in the reference management software used during manuscript preparation. This led to mismatched author names, journal titles, and publication details.

Below we provide the corrected information for each affected reference. Readers can match these corrections with the numbering of the original article to locate the accurate citations.

All corrected references are now authentic, traceable, and aligned with the content of the Review. The errors were unintentional and resulted from a reference management software malfunction. The authors sincerely apologize for the confusion caused and appreciate the opportunity to amend the scientific record.

## Corrected References

**Reference 1:** Zhao, C.; Xu, J.; Bi, H.; Shang, Y.; Shao, Q. A slow-release fertilizer of urea prepared via biochar-coating with nano-SiO_2_-starch-poly­(vinyl alcohol): Formulation and release simulation. Environ. Technol. Innov. 2023, 32, 103264. 10.1016/j.eti.2023.103264

**Reference 2:** An, C.; Sun, C.; Li, N.; et al. Nanomaterials and nanotechnology for the delivery of agrochemicals: strategies toward sustainable agriculture. J. Nanobiotechnol. 2022, 20 (11), 1–20. 10.1186/s12951-021-01214-7 PMC872541734983545

**Reference 3:** Su, Y.; Ashworth, V.; Kim, C.; Adeleye, A. S.; et al. Delivery, uptake, fate, and transport of engineered nanoparticles in plants: a critical review and data analysis. Environ. Sci.: Nano 2019, 6, 2311–2331. 10.1039/C9EN00461K

**Reference 4:** Lawrencia, D.; Wong, S. K.; Low, D. Y. S.; et al. Controlled release fertilizers: A review on coating materials and mechanism of release. Plants 2021, 10 (2), 238. 10.3390/plants10020238 33530608 PMC7912041

**Reference 5:** Li, N.; Sun, C.; Jiang, J. Advances in controlled-release pesticide formulations with improved efficacy and targetability. J. Agric. Food Chem. 2021, 69, 12579–12597. 10.1021/acs.jafc.0c05431 34672558

**Reference 6:** Del Prado-Audelo, M. L.; Bernal-Chávez, S. A.; Gutiérrez-Ruíz, S. C.; et al. Stability phenomena associated with the development of polymer-based nanopesticides. Oxid. Med. Cell. Longev. 2022, 5766199. 10.1155/2022/5766199 35509832 PMC9060970

**Reference 7:** Lee, P.; Lin, X.; Khan, F.; Bennett, A. E.; Winter, J. O. Translating controlled release systems from biomedicine to agriculture. Front. Biomater. Sci. 2022, 1, 1011877. 10.3389/fbiom.2022.1011877

**Reference 8:** Guha, T.; Gopal, G.; Kundu, R.; Mukherjee, A. Nanocomposites for delivering agrochemicals: a comprehensive review. J. Agric. Food Chem. 2020, 68 (12), 3691–3702. 10.1021/acs.jafc.9b06982 32129992

**Reference 9:** Kumah, E. A.; Fopa, R. D.; Harati, S.; et al. Human and environmental impacts of nanoparticles: a scoping review of the current literature. BMC Public Health 2023, 23, 1059. 10.1186/s12889-023-15958-4 37268899 PMC10239112

**Reference 10:** Pirzada, T.; Farias, B. V.; Mathew, R.; et al. Recent advances in biodegradable matrices for active ingredient release in crop protection: toward attaining sustainability in agriculture. Curr. Opin. Colloid Interface Sci. 2020, 48, 121–136. 10.1016/j.cocis.2020.08.011 33013179 PMC7509166

**Reference 11:** Zhou, J.; Liu, G. Z.; et al. Stimuli-responsive pesticide carriers based on porous nanomaterials: A review. Chem. Eng. J. 2023, 455, 140167. 10.1016/j.cej.2022.140167

**Reference 12:** Beig, B.; Niazi, M. B. K.; Sher, F.; et al. Nanotechnology-based controlled release of sustainable fertilizers: a review. Environ. Chem. Lett. 2022, 20, 2709–2726. 10.1007/s10311-022-01409-w

**Reference 13:** Yin, J.; Su, X.; Yan, S.; Shen, J. Multifunctional nanoparticles and nanopesticides in agricultural application. Nanomaterials 2023, 13, 1255. 10.3390/nano13071255 37049348 PMC10096623

**Reference 14:** Beckers, S. J.; Staal, A. H. J.; Rosenauer, C.; Srinivas, M.; Landfester, K.; Wurm, F. R. Targeted drug delivery for sustainable crop protection: transport and stability of polymeric nanocarriers in plants. Adv. Sci. 2021, 8, 2100067. 10.1002/advs.202100067 PMC818820634105269

**Reference 15:** Bouhadi, M.; Javed, Q.; Jakubus, M.; et al. Nanoparticles for sustainable agriculture: assessment of benefits and risks. Agronomy 2025, 15, 1131. 10.3390/agronomy15051131

**Reference 17:** Moher, D.; Liberati, A.; Tetzlaff, J.; Altman, D.G. Preferred reporting items for systematic reviews and meta-analyses: The PRISMA statement. PLoS Med. 2009, 6 (7), e1000097. 10.1371/journal.pmed.1000097 19621072 PMC2707599

**Reference 18:** Veginadu, P.; Calache, H.; Gussy, M.; et al. An overview of methodological approaches in systematic reviews. J. Evid. Based Med. 2022, 15, 39–54. 10.1111/jebm.12468 35416433 PMC9322259

**Reference 20:** Singh, M.; Goswami, S. P.; Ranjitha, G.; et al. Nanotech for fertilizers and nutrients – improving nutrient use efficiency with nanoenabled fertilizers. J. Exp. Agric. Int. 2024, 46 (5), 220–247. 10.9734/jeai/2024/v46i52372

**Reference 21:** Zhai, X.; Li, Z. Remediation of multiple heavy metal-contaminated soil through the combination of soil washing and in situ immobilization. Sci. Total Environ. 2018, 635, 92–94. 10.1016/j.scitotenv.2018.04.119 29660731

**Reference 26:** Fellet, G.; Pilotto, L.; Marchiol, L.; Braidot, E. Tools for nanoenabled agriculture: fertilizers based on calcium phosphate, silicon, and chitosan nanostructures. Agronomy 2021, 11 (6), 613–625. 10.3390/agronomy11061239

**Reference 52:** Fincheira, P.; Hoffmann, N.; Tortella, G.; et al. Eco-efficient systems based on nanocarriers for the controlled release of fertilizers and pesticides: toward smart agriculture. Nanomaterials 2023, 13, 1978. 10.3390/nano13131978 37446494 PMC10343595

**Reference 60:** Yamini, V.; Shanmugam, V.; Rameshpathy, M.; et al. Environmental effects and interaction of nanoparticles on beneficial soil microorganisms. Environ. Res. 2023, 236, 116776. 10.1016/j.envres.2023.116776 37517486

**Reference 61:** Devasena, T.; Kumar, R.; Baskaran, K.; et al. Insights on the dynamics and toxicity of nanoparticles in environmental matrices. Bioinorg. Chem. Appl. 2022, 4348149. 10.1155/2022/4348149 35959228 PMC9357770

**Reference 74:** Rouphael, Y.; Colla, G. Toward sustainable agriculture through biostimulants. Agronomy 2020, 10, 1461. 10.3390/agronomy10101461

**Reference 75:** Sun, W.; Shahrajabian, M. H. Arbuscular mycorrhizal fungi as microbial biostimulants. Plants 2023, 12, 1731. 10.3390/plants12173101 37687348 PMC10490045

**Reference 79:** Rouphael, Y.; Colla, G. Synergistic Biostimulatory Action: Designing the next generation of plant biostimulants. Front. Plant Sci. 2018, 9, 1655. 10.3389/fpls.2018.01655 30483300 PMC6243119

**Reference 80:** Garg, S.; Nain, P.; Kumar, A.; et al. Next generation plant biostimulants and genome sequencing strategies for sustainable agriculture development. Front. Microbiol. 2024, 15, 1439561. 10.3389/fmicb.2024.1439561 39104588 PMC11299335

